# The Puddingless Patients
*On the ground of expense, as we are informed, puddings, eggs, and vegetables, are being cut off at a London hospital. The nursing staff the patients, and many of the doctors regret it.—Ed. *T. H.*


**Published:** 1888-07-21

**Authors:** 


### THE PUDDINGLESS PATIENTS.*

Hark to the wail of a puddingless man!
Sick and sorry, and pale and wan;
Meat is loathsome—takes long to eat;
And yet, say the doctors, nothing but meat.
Expenses are great, the puddings must go,
And eggs and green vegetables follow also;
Two slices of meat and a tater dry—
On this must the sick man flourish or die.
Oh! hark to the wail of a puddingless man!

Here is a girl, with starvation writ
On her poor pale face, yet never a bit
Of rice or batter, so easy to eat,
Shall follow her portion of tough dry meat.
Samaritans give her both sugar and tea,
Or sad were the fate she would have to dree.
The hospital gives her one meal a-day—
A meal without pudding!—her hunger to stay.
Oh! hark to the wail of a puddingless girl!

In the children's ward you can hear a cry
As no nurse with a pudding comes passing by:
Puddings are sweet, fit food for the young:
Oh, why was economy here begun?
We could dock our wine, let our curate go,
Do without seeing a prince, also;
But nourishment's more than medicine, say we,
And puddings and eggs our meed should be.
Oh! hark to the wail of a puddingless child!

* On the ground of expense, as we are informed, puddings, eggs, and vegetables, are being cut off at a London hospital. The nursing staff the patients, and many of the doctors regret it.—ED. *T. H.*

